# Part Meat and Part Plant: Are Hybrid Meat Products Fad or Future?

**DOI:** 10.3390/foods9121888

**Published:** 2020-12-17

**Authors:** Simona Grasso, Sylvia Jaworska

**Affiliations:** 1 Institute of Food, Nutrition and Health (IFNH), School of Agriculture Policy and Development, University of Reading, Reading RG6 6EU, UK; 2 Department of English Language and Applied Linguistics, University of Reading, Reading RG6 6AH, UK; s.jaworska@reading.ac.uk

**Keywords:** meat product, consumers, flexitarianism, corpus linguistics, online product reviews

## Abstract

There is a growing interest in flexitarian diets, which has resulted in the commercialisation of new hybrid meat products, containing both meat and plant-based ingredients. Consumer attitudes towards hybrid meat products have not been explored, and it is not clear which factors could affect the success of such products. This study is the first to overview of the UK hybrid meat product market and to explore consumer’s attitudes towards hybrid meat products in 201 online reviews, using tools and techniques of corpus linguistics (language analysis). In the positive reviews, consumers emphasised the taste dimension of the hybrid meat products, seeing them as healthier options with good texture and easy to prepare. The negative reviews related to the poor sensory quality and not to the concept of hybridity itself. Using a multidisciplinary approach, our findings revealed valuable insights into consumer attitudes and highlighted factors to consider to market new hybrid meat products effectively.

## 1. Introduction

High levels of meat consumption are associated with perceived health, social and environmental concerns resulting in calls to reduce the quantity of meat we consume [1]. To achieve a partial substitution of animal proteins in the diet with more sustainable plant proteins, long-term dietary transitions rather than short phases need to be established [2].

Studies have found that to create an effective dietary change, new practices should not diverge too much from consumers’ previous behaviour [3]. Food choice has been recognised as a complex process that goes beyond sensory properties and involves many factors that can be grouped into the characteristics of the consumer, the product and the specific context in which the choice is made [4]. Factors related to consumer behaviour, which might limit consumer transition to alternative protein sources, are convenience and minimal cooking skills [5].

It is difficult to fully shift from a meat-centric diet to strict vegetarianism or veganism because of positive beliefs and attachments to meat and meat-centric societal constructs, however switching to a flexitarian or semi-vegetarian diet (mainly plant-based, with limited meat consumption) is less strict and can still have a positive impact [6].

A survey by the Humane Research Council [7] on 11,399 Americans found that 5 out of 6 people who become vegans or vegetarians eventually went back to eating meat. The authors suggest that it would be more important to persuade the majority of the population to reduce meat consumption rather than convincing a small percentage to give up meat completely [8].

Flexitarianism is increasing in popularity amongst consumers, with a market research study in the UK [9] reporting that while around 90% of consumers eat red meat or poultry, more than a third (34%) of eaters and buyers of meat and poultry have regular days when they avoid meat.

Within this context, the concept of hybrid meat products, that is meat products in which a proportion of meat has been partially replaced by more sustainable protein sources, might be suitable to bridge the gap between meat and meat-free products, while providing convenience, and allowing consumers to continue using foods as they would conventionally do [10].

Hybrid meat products could open new business opportunities for the food industry [11], and indeed very recently, hybrid meat products have started appearing in the UK market [12]. The meat industry might be answering the growing flexitarian consumer needs, but the launch of hybrid meat products might also be representing a moment for change and an attempt from meat manufacturers to gain additional market share over new popular plant-based alternative protein sources [8]. As Hicks et al. [13] point out, “it would be efficient and wise for the meat industry to build a strategy around the flexitarian demographic, to ensure their needs are met and to keep them consuming meat, rather than risk losing them to veganism”.

We will now discuss the difference between hybrid meat products and meat extenders, provide a literature overview on consumer attitudes towards hybrid meat products and introduce the corpus linguistics (language analysis) techniques that will help achieve the aims of the current study.

Many processed meat products available in the market are already somehow “hybrid” as they often do not contain 100% meat [8]. For example in the UK, according to the Meat Products Regulation [14], only 42% of pork is needed to label sausages as pork sausages and the pork meat used can contain 30% fat and 25% connective tissue. A variety of functional ingredients have been traditionally added to processed meats, including fillers (plant substances with high carbohydrate content), extenders (non-meat compounds with considerable protein content), and binders (substances with high-protein content able to bind both water and fat) [15]. Indeed, plant-based ingredients from soy and wheat have been used by the meat industry to achieve cost savings [16], as well as for their functional properties: fat emulsification, gelling capability, and water binding [17].

The difference between hybrid meat products and meat products with plant-based functional ingredients (extenders, fillers and binders) is in the purpose of the mix of meat and plant proteins [8]. Usually plant-based functional ingredients are used traditionally for economic and technological reasons, in hybrid meat products this concept is pushed further to include positive connotations on the meat “extension”, including healthiness, lower environmental impact and generally the idea of decreasing meat consumption [8].

Several research articles have shown that although challenging, it is technologically feasible to manufacture hybrid meat products such as burgers, meatballs and sausages with acceptable sensory quality [10,11,18,19]. However, consumer attitudes towards hybrid meat products have been investigated in a limited number of studies [8]. A study by de Boer et al. [20] compared hybrid meat products vs. alternative protein snacks such as insects, lentils and seaweed. The most popular snack was the hybrid one (chosen by 54% of 1083 participants). The authors concluded that it would be valuable to combine animal and plant-based protein and that hybrid meat products could be acceptable to lowly involved consumers who will not actively search for more environmentally friendly proteins. Similarly, previous work by the same authors found that hybrid meat products could be acceptable to many consumers, especially those who are weakly involved, because they may seem more familiar to them [5,8].

While these studies offer initial invaluable findings that could be used to develop more popular hybrid meat products, more research is still needed to understand sensory aspects and specifically consumer attitudes towards those products. Having a better understanding of which factors might have an impact on consumer acceptability would allow the effective formulation and marketing of existing and future hybrid meat products. A more holistic and multidisciplinary approach could offer richer and more nuanced insights into the stance and views of consumers, including a range of perceived advantages and disadvantages. Consumers’ online reviews provide a unique opportunity to do so and allow the researcher to tap into consumers’ authentic responses and opinions on dimensions that are relevant to them, but might not have been included and tested in previous research. Because online reviews are essentially texts, they require text analytics derived from linguistics. We explored consumer’s attitudes towards hybrid-meat products in online reviews by utilising tools and techniques of corpus linguistics that allowed quantitative identification of the most frequent words and key terms across larger textual data sets. Frequency counts and key terms are useful in that they can highlight the distinctive (salient) words and two-word combinations in a given data set (a corpus of texts), which in turn point to dominant stances and attitudes shared by producers of the texts. The tools and techniques of corpus linguistics were applied to study a corpus of 201 online reviews in order to identify the dominant stances and opinions expressed by consumers who bought and consumed hybrid-meat products.

The aim of this study was therefore to (1) review the presence of hybrid meat products in the UK market, and (2) extract UK online consumer reviews on hybrid meat products and gather preliminary consumer insights utilising tools and techniques of corpus linguistics.

## 2. Materials and Methods

### 2.1. Hybrid Meat Products in the UK Market and Review Collection

A web search was conducted to understand the presence of hybrid meat products in the UK market. If a specific retailer was found to have launched a range, then the retailer website was further investigated to get more details on the characteristics and availability of each product. Product details were compiled into a list. Some of these products contained customer product reviews, and all available reviews were collected. This made up a total of 201 reviews from the websites of Waitrose, Ocado and Sainsbury’s for three hybrid meat products. The products were Waitrose pork, chickpea and spinach sausages (79 reviews), Waitrose harissa chicken cauliflower rice & chickpea meatballs (106 reviews) and Sainsbury’s Love Meat & Veg! Mediterranean beef meatballs (16 reviews). All reviews are publicly available on the retailer’s website and were downloaded into an excel spreadsheet, noting down the product name, review title, review comment and score from 1 to 5. The reviews were divided into two groups: reviews with a score equal to or above 3.5 were included in the corpus of positive reviews, whereas reviews with a score below 3.5 and below formed the corpus of negative reviews. Table 1 shows the size of the two corpora, with the majority of reviews being positive (80%).

Because the reviews were short and often included only a few words (not complete sentences), the sizes of the corpora are relatively small. Nonetheless, they were still large enough to perform frequency counts and key term analysis.

### 2.2. Statistical Approach for Frequency Counts and Key Term Analysis

Both positive and negative corpora were uploaded onto the linguistic software program Sketch Engine, which performed frequency counts and an extraction of key terms. Frequency counts of language items in reviews can be a useful indicator of preferences in that frequent items can signal preferred lexical choices which in turn can point to attitudes and stances. Yet, the most frequent items in English are grammatical words (e.g., articles, prepositions) that as such are used to form grammatical constructions and do not hold a lexical meaning. Because we were interested in attitudes towards the hybrid-meat products, and attitudes are likely to be revealed in the ways in which the consumers describe and evaluate the products, the analysis was focused on adjectives. Adjectives are parts of speech that function primarily as descriptors denoting a whole range of features and dimensions such as size, colour, quantity, texture, taste, judgment and affect [21]. Since all these dimensions can be relevant to hybrid-meat products, adjectives were selected as good indicators of consumers’ attitudes towards specific features of the products.

Once the corpora were uploaded onto Sketch Engine, a parser was applied which tagged each word in a given corpus with its parts of speech (verbs, nouns, adjectives, etc.). In this way, adjectives were identified, and their frequencies retrieved. Because adjectives can describe a range of dimensions, subsequently all adjectives retrieved from the two corpora were grouped into their semantic domains. This allowed us to determine which dimensions of the products (e.g., texture, taste) were particularly emphasised and how they were evaluated by those who liked and disliked them. The grouping of adjectives into semantic domains was conducted first independently by the two researchers; disagreements and ambiguous meanings (e.g., the adjective ‘hot’ can be used to describe temperature or the level of spiciness) were resolved by checking the meanings of the adjectives in context, that is, how they were used in the reviews.

In order to gain insights into other salient themes and issues mentioned by the consumers, we also retrieved distinctive multiword items from both corpora, also known as key terms. Key terms are simply distinctive combinations of two or three words which appear more frequently in the studied corpus as compared to a reference corpus and, additionally, match the typical format of terminology in the language, that is, they are lemmatised. Key terms are good indicators of the content and distinctive topics of the studied corpus. For the purpose of this analysis, we used the EnglishTenTen corpus (available on Sketch Engine) as a reference corpus because it is a large compilation of general English collected from online sources. The key terms were retrieved using keyness scores calculated as follows:

$$\frac{fpm_{focus}+n}{fpm_{ref}+n}$$

*Fpm_focus_* stands for normalised frequency (per million) of the term in the focus corpus (in our case in positive or negative reviews), while *fpm_ref_* is the normalised frequency (per million) of the term in the reference corpus. *N* is the simple maths parameter added to account for the problem that we cannot divide by zero. Retrieved key terms were then grouped into semantic domains using the same procedure as above. The next section summarises the main findings that emerged from the search of hybrid meat products in the UK market and the results of the corpus linguistic analysis.

## 3. Results and Discussion

### 3.1. Hybrid Meat Products in the UK Market

Table 2 shows a list of hybrid meat products launched in the UK with information on the brand, launch date, range, prices, availability and percentage of plant-based ingredients used in the formulation (where available). Hybrid meat products were launched in four UK supermarkets (Marks and Spencer, Waitrose, Tesco, Sainsbury’s and Aldi) between 2017 and 2020. More details on each retailer launch are discussed here.

According to our search, in 2017 Aldi was the first UK retailer to launch an own-label hybrid meat product. The retailer launched the flexitarian “Full of Beans” chilled mince in 2017 [22] and a burger with haricot beans. Because of its flexitarian name, the burger attracted negative press and social media attention [23], and neither of the two products are currently available on the retailer’s website. It is interesting to note that the plain red packaging used by Aldi did not convey any special message regarding the non-meat ingredients, it just highlighted the characteristics of the meat component “Scotch BBQ flexitarian burgers-reared to higher welfare standards from farms we know and trust”.

Waitrose and Sainsbury’s both launched their hybrid meat product ranges in 2018 (Table 2). Waitrose, a UK retailer targeting upper-middle class consumers, launched a range of 9 hybrid meat products with 20–50% vegetables in 2018 and at least 3 of them are still available for purchase on the retailer website today [24]. The sausages were developed “for shoppers looking to reduce their meat intake” and carry the green Waitrose ‘Good Health’ label, designed to make it easier for shoppers to make healthier choices. Sainsbury’s in 2018 launched a range called “Love Meat and Veg”. The range aimed to help consumers to reduce their meat-eating habits and explore the switch to a higher vegetable intake targeting flexitarians. They contain 50% meat and 50% vegetables and include the range shown in Table 2. However, on the Sainsbury’s website, only Mediterranean Beef Meatballs seem to be currently available. Both of these retailers made the ranges look different from the meat versions and plant-based version, using colourful packaging with vegetables and attractive names and these are all factors that have been shown to affect food choice [4].

Tesco in 2019 introduced the “Meat & Veg” range [25] which comprises products made from beef or lamb. The retailer claims that this range “helps make scratch cooking easier, removing the need to buy vegetables separately to make the base of popular dishes such as bolognese, lasagne or meatballs” and “the range champions vegetables as flavour enhancers to provide sweetness to home-cooked dishes”. The focus of these products seems to be on delivering convenience and flavour to consumers, rather than highlighting the lower meat content or the health characteristics. All the products in the range are currently available to purchase on the Tesco website.

The last retailer to launch hybrid meat product is Marks and Spencer [26]. The range includes 3 products, and they have been formulated to deliver “1 of your 5 a day” per portion. Here the focus is on vegetables as healthy ingredients but also as flavourful compounds (“an easy way to your five a day” and “more veggies, more flavour”).

A total of 4 private labels also launched hybrid meat products into the UK market between 2016 and 2017. These were ABP Food Group under the Debbie & Andrew’s brand, Walkers Sausage Co. under the brand MOR sausages, Kerry under the brand The Crafty Carnivore and Finnerbrogue Artisan under the brand #Funky Flexitarian. Debbie & Andrew’s launched the range “Flexilicious” in 2017 [27]. The flexilicious sausages consisted of 40% beef, 40% vegetables and legumes, 10% herbs and seasonings along with 10% gluten-free crumbs and water. They launched on Amazon Fresh, Asda, Morrisons, Ocado, Sainsbury’s and Tesco, but they were not available for purchase when our search was conducted. MOR sausages launched in 2017 [28] and contained 52–60% meat and the inclusion of a variety of vegetables. Although the website suggests that MOR sausages are stocked in Tesco and Morrisons, these products were not found on the retailers’ websites. Kerry’s The Crafty Carnivore range of 2 sausages and Finnerbrogue Artisan’s #Funky Flexitarian range of 4 sausages launched in 2016 were not successful either.

Finally, in the foodservice sector, 2 examples of hybrid meat products are available. Byron burger, a chain with 53 stores in the UK, launched the “Classic Flex” in 2018, made of 70% British beef and 30% mushrooms [29], however, this item is not on the current menu. In October 2019, Brew Dog launched a patty with 50% UK beef and 50% plant-based “Beyond Meat” [30], however, this was not on the online menu, and it was only offered as a “special” for the month of October.

In total, 38 hybrid meat products were launched in the UK in 2016–20, and 12 of these products seem to be still currently available for purchase [8]. These numbers should not surprise, as it is well known that most new foods fail in the market [31]. The most popular hybrid meat products launched onto the market were sausages, with 20 products launched, followed by meatballs with 7 launches, burgers with 6 launches and mince with 5 launches. The base meats used vary: beef and pork were the most popular (16 and 15 products respectively), followed by chicken (5 products) and lamb (2 products). The amount of meat to non-meat ingredient ratio changes widely, from 25% of vegetables, up to 50% of vegetables. The type of non-meat ingredients used also vary, and they are usually a blend of different spices, fruits and vegetables, however, mushrooms have also been used as an ingredient on their own, as mushrooms have been shown to be effective in maximising umami taste in meat formulations [32].

Interest in processed meat products with plant-based ingredients is increasing, with Mintel [12] reporting in a recent survey on 1678 consumers that on average 27% of processed meat buyers would be interested in buying processed meat products with added vegetables. This number increases to 35% for consumers who eat meatballs and to 31% for consumers who eat burgers.

However, looking at the launch dates and current availability in the market, this search shows that so far, attempts to bring to the market hybrid meat products have had mixed results. Some of the earlier launches did not seem to have managed to maintain a place in the market, and perhaps they might have been received with confusion or were not understood by consumers. The newer retailer launches by Tesco, Sainsbury’s, Waitrose and Marks and Spencer, carry a more targeted message to consumers [8]. Overall, it seems that the most recent launches do not mention flexitarianism and stress more the flavour, healthiness and convenience of these meat products, including messages such as “5-a-day”, the convenience of having vegetables already in minced meat and the use of vegetables as flavour enhancers. For these new launches to be successful, it is important to codevelop and codesign new foods with consumers, and the new product development literature stresses the importance of incorporating consumer insights into the new product development process for foods [31].

### 3.2. Consumer Attitudes and Evaluations in Hybrid Meat Reviews

Table 3 shows all the adjectives identified and retrieved from the corpus of positive reviews and their semantic classification. As can be seen, consumers emphasised mostly the taste dimension of hybrid meat products. Taste is quite a subjective domain, but many of those who purchased the products evaluated them as tasty, delicious and spicy. These results are in accordance with those by Reed et al. [33], who found that in commercial food product reviews, taste-associated words were mentioned more than words associated with other factors such as price, food texture, customer service, nutrition or smell. After taste, there was a heavy use of general positive descriptors, which is not surprising given the positive scores. Interestingly, several consumers saw the products as healthier options (19 mentions in total), low in fat (mentioned in 3 reviews) and as a way to increase the intake of vegetables (mentioned in 7 reviews). It can therefore be deduced that the hybrid meat products are appreciated by consumers who are health conscious. This is an interesting finding related to hybrid meats, as traditional meat products in the literature have scored quite low in terms of perceived healthiness by consumers [34]. Consumers also seem to positively value the texture of the products and their quick and easy preparation at home. Processed meats can be a convenient type of food product [31] and Grunert [35] suggested that there might be a synergy between the desire for healthiness and the demand for convenience in functional food products.

A total of 4 positive reviews included negative adjectives such as ‘sceptical’ and ‘suspicious’, but these were to emphasise consumers’ initial suspicions which disappeared once the products were tasted. The following extracts are indicative of this stance:

(1) “Bought off the back of good reviews, was a bit sceptical that a meatball with cauliflower, etc. would actually be nice but I thought these were superb”.
(2) “When I put these in the pan I was sceptical, because the cauliflower smell was very strong. As it happens these are tasty and quite light for a meatball”.

These four reviews can be related to the concepts of willingness to try, change-seeking and consumer innovativeness as a way to relate to new foods [36], which in this case led to a positive outcome.

The top key terms revealed similar themes. The largest category was two-word combinations highlighting the healthiness of the products, with 19 mentions in total (see Table 4). Consumers seem to view hybrid meats as a healthy choice, as a way to reduce meat consumption and increase the intake of vegetables. The following extract from the corpus are indicative of this stance:

(3) “These sausages tasted good and are a great way to get extra veg into the kids”.
(4) “We love that we can enjoy healthy choice sausages”.
(5) “Healthy sausage, pleased with these sausages as lower in fat but still tasty!”.

The positive reviews indicate that hybrid meats might be seen as an opportunity to lower meat intake and increase vegetable intake while maintaining acceptable taste (as seen in Table 3). It has been reported in the literature that consumers are unwilling to compromise on taste when it comes to healthier food options [37], therefore, hybrid meat products should be designed to deliver both on taste and health.

The second most distinctive domain was that of novelty and positive surprise. Many consumers who took to the online forum to review the products emphasised that this was something new in their shopping basket almost treated as a form of experiment, which met the expectations:

(6) “It was with a pleasant surprise with all the veg how juicy they where (sic) and very tasty great combination of flavours very good and something a little different from standard”,
(7) “A tasty change found this to be a delicious change from ordinary meatballs”.
(8) “This is the first time we purchased this sausage will certainly be purchasing it again”.
(9) “Interesting new combination tasty new experiment, hopefully will continue to be stocked”.

These reviews highlight the interest of these consumers in purchasing something new and show how the differentiation strategy in these hybrid meat products were well received by consumers.

Consumers also emphasised that the sausages were enjoyed by the whole family, which made the hybrid meat products a convenient alternative.

In terms of the negative reviews, it is not surprising to find the category of negative descriptors as the most prominent ones (see Table 5). The dissatisfaction with the products seemed to be mostly due to a lack of taste and texture as evidenced with the use of adjectives such as ‘flavourless’, ‘tasteless’, ‘bland’, ‘mushy’ and ‘dry’. It is therefore not necessarily the concept of hybridity itself but rather the specificity of the product’s sensory quality that did not seem to match expectations. Consumers purchased the hybrid-meat products, which suggests that there was a willingness to try these new foods because they were perceived as healthy.

However, the experience proved to be disappointing; the extracts below exemplify this trend in the negative online reviews:

(1) “Thought I’d give these meatballs a try as they seemed a bit different. But I found them disappointing because they fell apart when cooking and not very tasty”.
(2) “Tried twice to check, still awful & flavourless”.
(3) “Trying to find healthy food for kids. Unfortunately I had to throw away because the texture was so unpleasant, slimy and rubbery”.
(4) “OK because nice to know some veg in there but my husband and I werent (sic) keen on taste”.

The analysis of key terms confirms the dominant-negative experience of the taste and texture of the products in the negative online reviews (Table 6). Yet, because of the small sample under consideration (only 40 reviews), it is difficult to generalise from these results. Overall, we can say that both the positive and negative reviews on taste highlight the paramount importance of this sensory attribute.

It is worth pointing out the limitations of this study. The linguistic analyses were carried out on a limited sample of reviews, and further analysis should be conducted on a larger sample. The negative reviews were lower in number compared to the positive one. This could be because genuinely, most of the consumers who tried the products had a positive view on them, however, it is possible that consumers with negative views did not report them online. In addition, many who purchased the hybrid meat products might have decided not to leave a review at all, therefore, the online product reviews we have gathered cannot be used on their own to make specific recommendations on these products.

## 4. Conclusion and Future Work

This study was the first investigation into commercially available UK hybrid meat products, and the first preliminary exploration into consumers’ attitudes towards hybrid meat products using online product reviews. The messages adopted by retailers to promote hybrid meat products have changed greatly since these products were first introduced to the market. The linguistic analysis showed that the most important themes related to hybrid meats were taste, followed by healthiness and convenience. There are still several gaps in the literature that need to be investigated. Future research should include both qualitative and quantitate studies developing the topic of hybrid meat products and further the understanding of its potential. It would be valuable to compare different socio-demographic characteristics and different nations to gather social and cross-country insights on this topic. For hybrid meat products not to be a fad, product development needs to be codesigned with consumers and carried out by teams of multidisciplinary professionals investigating recipe reformulations, sensory aspects and consumer attitudes simultaneously, in a more holistic approach.

## Figures and Tables

**Table 1:** Size of the corpora of online product reviews on three UK hybrid meat products.

Corpus	No. and % of Reviews	No. of Words
Positive reviews	161 (80%)	4223
Negative reviews	40 (20%)	1148

**Table 2:** The brand, launch date, range, prices, availability and % of plant-based ingredients used in hybrid meat products launched in the UK.

Brand	Launch Date	Range Launched and Prices	Product Still Available?	% of Plant-Based Ingredients Used (If Not on Product Name Already)
Retailer brand—Marks and Spencer	6 Jan 2020	Hidden Veggies range:		Hidden Veggies range: one of your five a day per portion (vegetable content unknown)
		Beef and carrot mince (£3.50)	Yes	
		Hidden Veggies chicken and vegetable meatballs	Yes	
		Hidden Veggies beef and mushroom burgers	Yes	
Retailer brand—Tesco	April 2019	Meat & Veg range:		
		Tesco Meat & Veg 4 Beef, Carrot & Onion Burgers (454 g/£2.50)	Yes	35% average vegetable blend: vegetable blend (38%) of carrot and onion
		Tesco Meat & Veg 8 Beef, Red Pepper & Carrot Koftas (600 g/£3.00)	Yes	vegetable blend (30%) of carrot, onion, red pepper
		Tesco Meat & Veg Lamb, Carrot & Onion Mince (500 g/£4.00)	Yes	vegetable blend (38%) of carrot and onion
		Tesco Meat & Veg 12 Beef, Carrot & Onion Meatballs (336 g/£4.00)	Yes	vegetable blend (31%) of carrot, onion, butternut squash
		Tesco Meat & Veg Lean Beef, Carrot and Onion Mince (250 g/£2.19, 500 g/£3.39, 750 g/£4.50)	Yes	vegetable blend (31%) of carrot, onion, butternut squash
Retailer brand—Waitrose	10 Jan 2018	6 Pork, Butternut Squash, Quinoa & Kale Sausages £3.29/400 g	No	25% vegetables
		12 mini Pork, Butterbean, Lentil & Garlic Toulouse Style Sausages £3.29/400 g	Yes	30% vegetables
		6 Spanish Style Pork, Chickpea, Spinach & Tomato Sausages £3.29/400 g	Yes	35% vegetables
		12 Harissa Chicken, Cauliflower Rice & Chickpea Meatballs £3.29/360 g	Yes	25% vegetables
		20 Asian-Style Beef Meatballs with Beans £3.29/300 g	No	35% vegetables
		2 Caribbean Inspired/Style Spiced Pork, Sweet Potato & Black Turtle Bean Burgers £3.29/270 g	No	Unknown
		Waitrose Cumberland Chipolata with Mixed Pulses £3.29/375 g	No	20% vegetables
		Waitrose Beef Meatballs with Mixed Pulses £2.49/300 g	No	50% vegetables
		Waitrose Beef Mince with Mixed Pulses £3.99/454 g	No	50% vegetables
Retailer brand—Sainsbury’s	early 2018	Sainsbury’s Love Meat and Veg range all £2.50/350 g pack:		50% meat and 50% vegetables
		Mediterranean Beef Meatballs	Yes	
		Chicken Sausages with Feta, Spinach and Peas	No	
		Pork Sausages with Kale and Butternut Squash	No	
		Pork Sausages with Kidney Beans, Sweet Potato and Smoked Paprika	No	
		Pork Sausages with Roasted Red Pepper, Sundried Tomato and Quinoa	No	
Retailer brand—Aldi	early 2017	Flexitarian “Full of Beans” chilled mince (£1.99/400 g)	No	50% (haricot beans)
		BBQ Flexitarian burger	No	
Private label—Finnerbrogue Artisan	2016	#Funky Flexitarian range:		47% vegetables and legumes
		Spicy lamb’alafal chipolatas	No	
		Smokey pork n’bombay beet bangers	No	
		Lightly curried cauli’nation chicken chipolatas	No	
		Beef, tomato n’basil bangers	No	
Private label—Debbie & Andrew’s brand (ABP group)	Jan 2017	Flexilicious range:		40% vegetables and legumes
		Chilli Con Carne Beef Sausages	No	
		Super Sausages 6 Chorizo Style Pork & Bean 400 g	No	
Private label—MOR Sausages	2017	Moroccan Spiced Pork & Red Pepper Sausages	No	% of plant-based ingredients not specified: Pork (55%)
		Mediterranean Chicken with Sundried Tomato & Basil Chipolatas	No	Chicken (60%)
		Pork, Super Green Veg & Lentil Sausages	No	Pork (55%)
		Pork, Beetroot & Bramley Apple Sausages	No	Pork (52%)
Private label—Kerry	2017	The Crafty Carnivore range:		43% vegetables and legumes
		Smoky Chipotle Pork Sausages with Sweet Potatoes and Red Pepper	No	
		Harissa Spiced Pork Sausages with Butternut Squash and Red Pepper	No	40% vegetables and legumes
Restaurant chain—BrewDog	October 2019	Burger	No—special in October only	50% plant-based “Beyond Meat”
Restaurant chain—Byron	2018	Classic Flex Burger	No	30% mushrooms

**Table 3:** Semantic domains of adjectives in positive online reviews.

Semantic Domain/Dimensions	Adjectives	Total Freq.
Taste	tasty (76), delicious (34), spicy (13), yummy (4), flavoursome (3), succulent (2), well-seasoned (2), strong (2), tangy (1), scrumptious (1), subtle (1), versatile (1)	140
General positive descriptors	great (39), good (21), nice (21), different (12), lovely (7), excellent (4), new (4), unusual (4), popular (4), perfect (2), pleasant (2), superb (2), traditional (2), wonderful (2), special (2)	128
Health	healthy (19), fresh (7), extra [veg] (5), low [in] (3), balanced (2)	36
Texture	moist (7), dry (5), meaty (4), fatty (2), mushy (2), soft (2), hard (2), light (2), tough (2)	28
Manner of processing	quick (7), easy (7), homemade (2), simple (2), standalone (1)	19
People	[e.g., my 3 year] old (4), whole (4)	8
Colour	brown (3), red [meat] (3), white (1)	7
Temperature of processing	cold (2), hot (2), warm (1)	5
General negative descriptors	sceptical (2), suspicious (1), unpleasant (1)	4
Portion size	small (2)	2

**Table 4:** Key terms in positive reviews.

Semantic Domain	Key Terms	Total Freq.
Health	right amount (3), healthy choice (2), meat consumption (2), veg content (2), red meat (2), healthy sausage (2), fat content (1), low meat content (1), healthy tasty dinner (1), healthy eating (1), nice balance (1), low carb (1)	19
Novelty/Surprise	first time (8), pleasant surprise (2), great alternative (1), standalone alternative (1), tasty alternative (1), delicious change (1), tasty change (1), tasty new experiment (1)	16
General positive terms	great way (3), regular addition (2), great find (2), great idea (2), excellent sausage (2), great combination (1)	14
Taste/texture	good flavour (3), great texture (3)	9
People	whole family (6)	6

**Table 5:** Semantic domains of adjectives in negative online reviews.

Semantic Domain/Dimensions	Adjectives	Total Freq.
Taste	spicy (3), flavourless (3), tasteless (2), bland (1), flavoursome (1), metallic (1), overpowering (1), salty (1), tasty (1), unseasoned (1)	36
General negative descriptors	disappointing (8), awful (5), unpleasant (2), inedible (2), poor (2), sloppy (2), strange (2), weird (2), bad (1), disgusting (1), dreadful (1), freaky (1), gross (1), horrible (1), nauseous (1), negative (1), underwhelming (1), uneatable (1)	35
Texture	mushy (4), dry (3), soft (3), rubbery (2), slimy (2), tough (2), chewy (1), crumbly (1), moist (1), pappy (1), solid (1), heavy (1)	25
General positive descriptors	good (5), amazing (1), pleasant (1), great (1)	8
Temperature of processing	hot (1), cold (1)	2
Health	healthy (1)	1
Manner of processing	undercooked (1)	1
People	fussy [eater] (1)	1
Colour	green (1)	1

**Table 6:** Key terms in negative reviews.

Semantic Domain	Key Terms	Total Freq.
Taste/texture	poor texture (2), metallic aftertaste (1), unseasoned chicken (1), lacked flavour (1), real flavour (1), recognisable flavour (1), awful smell (1), overpowering taste (1), freaky texture (1), slimy texture (1), strange texture (1), pappy thing (1), undercooked veg (1), dry side (1), sloppy mix (1)	16
General positive terms	good sauce (1), natural shape (1), plus side (1)	3
General negative terms	bad news (1), very poor (1)	2
People	single person (1), toddler wouldn’t (1)	2
Health	healthy food (1)	1
